# Liquid Crystal Elastomers: 30 Years After

**DOI:** 10.1021/acs.macromol.4c01997

**Published:** 2025-03-06

**Authors:** Eugene M. Terentjev

**Affiliations:** Cavendish Laboratory, Cambridge University, JJ Thomson Avenue, Cambridge CB3 0HE, U.K.

## Abstract

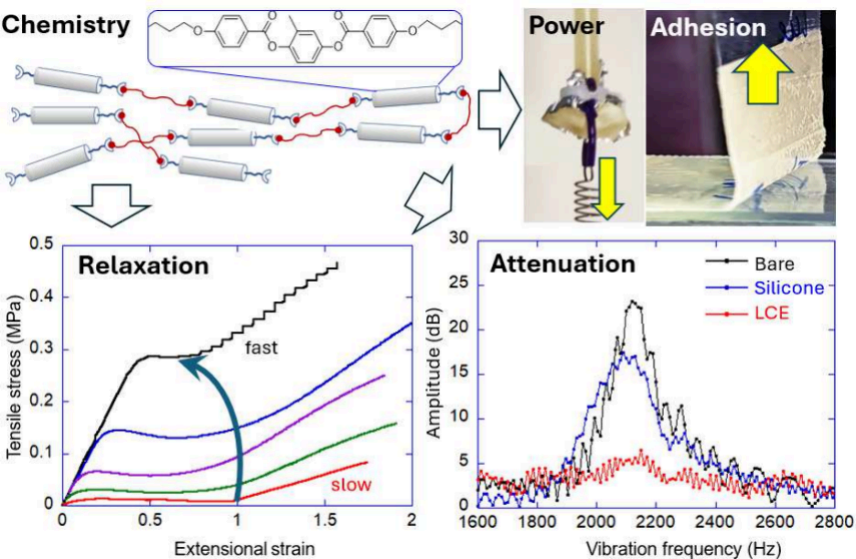

This is a Review that attempts to cast a look at the whole history of liquid crystal elastomers and the evolution of this field from its inception to the current state of the art. The exposition is limited by deliberately omitting several important elements of this field, such as densely cross-linked networks or smectic elastomers, focusing solely on the nematic phase of these elastomers. In this more narrow topic, we first discuss the current developments and perspectives in the materials chemistry. This is followed by three sections, each dedicated to one of the three main points of interest in the nematic liquid crystal elastomers: the reversible actuation, the soft elasticity, and the viscoelastic dynamics of nematic elastomers. In each of these directions, there have been significant developments over recent years but equally significant new avenues emerging for the research to follow.

## Introduction

“Smart materials” is a cliché used broadly in many areas of modern technology, in each case meaning different things. Materials that offer an active response to a stimulus are of particular interest in this context. Materials with properties such as shape memory,^1^ self-healing,^2^ affinity modulation,^3^ or shape change^4,5^ can push the limits of what traditional inorganic materials could offer. Among these are polymer actuators, which are materials capable of producing mechanical work in response to an external stimulus. Liquid crystal elastomer (LCE) materials represent examples of such “smart” systems, representing a new state of matter^6,7^ where the intrinsic phase transformations driven by molecular interactions are directly translated into the changes in the macroscopic shape of the material and its dynamic-mechanical characteristics. The ability to manipulate properties of liquid crystals by subtle chemical changes and the remarkable mechanical response of LCEs (with strain between 5% and 500%, stress up to 20 MPa, and the speed of response only limited by the rate of heat transfer) make them extremely promising systems for actuators and artificial muscles.

**Figure 1:**
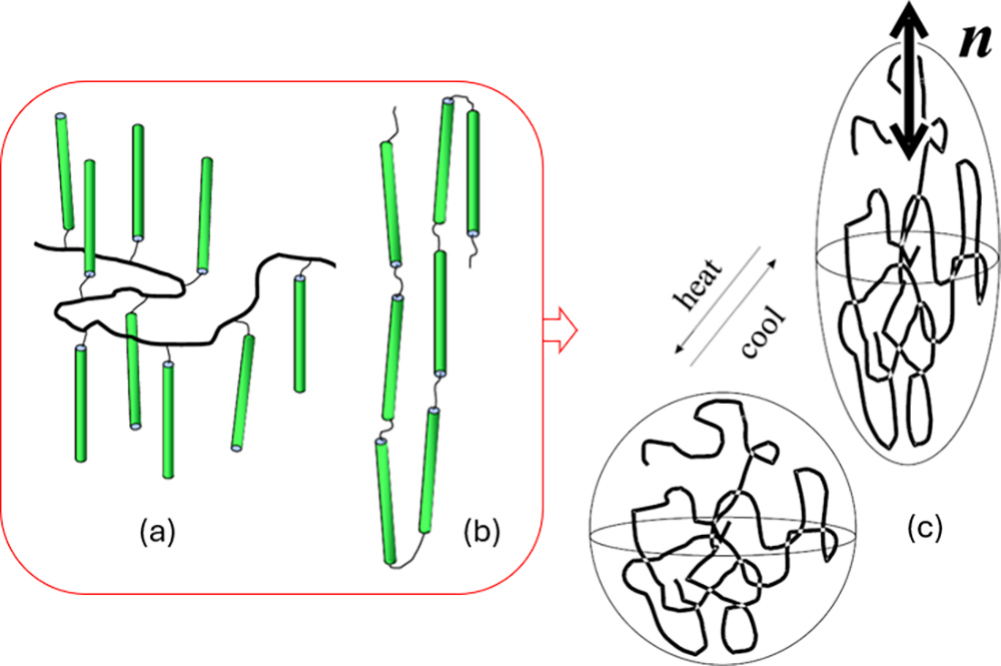
A schematic “topology” of the liquid-crystalline polymer that makes the LCE. There has to be either side-chain (a) or main-chain (b) attachment of rod-like mesogenic units, which are then cross-linked into a rubbery network. Since the length scale on which cross-links are established is much greater than the coherence length scale of the average mesogenic ordering, the rubber-elasticity deformation and the LC order parameter are independent (albeit coupled) physical fields. Specifically, in the uniaxial nematic LC phase, such a mesogenic polymer chain adopts an average asymmetric shape (c), which is prolate or oblate depending on the nature of the LC polymer.

The first LCEs were created in the 1980s by innovative polymer chemists: primarily Finkelmann,^8^ but also Shibaev and Talroze,^9^ Zentel,^10^ Keller,^11^ and Mitchell and Davies.^12^ In all early studies, the LCEs were formed by cross-linking the side-chain liquid-crystalline polymers, which combine rigid-rod mesogenic molecular groups with the backbone polymer chain in the configuration illustrated in Figure 1(a). In general, the backbone polymer chain was either polysiloxane, attaching the side groups via the hydrosilylation reaction, or polyacrylate formed by homopolymerization of acrylate-terminated mesogenic molecules. Rare examples of main-chain LCEs in the 1990s, in the topology illustrated in Figure 1(b), are those of Finkelmann^13^ and Percec,^14^ in both cases synthesizing divinyl-terminated rigid-rod mesogens and polymerizing them into chains that are subsequently cross-linked by hydrosilylation. Of note is an even earlier combined main-chain/side-chain topology from Zentel.^15^

The understanding that in a LCE one is dealing with a material conceptually different from classical elastic systems has been growing steadily, beginning from the pioneering study by De Gennes^16^ that preceded any experimental realization. Indeed, De Gennes had long anticipated that something remarkable must occur if one manages to create an elastic solid with sufficient internal mobility to permit spontaneous nematic orientational order. In 1988, Khokhlov^17^ and Warner^18^ produced almost identical early models of how the natural shape of the aligned LCE would change with changes in the order parameter: the modern understanding of LCE actuation is still resting on those ideas. For a short while, this developed into a competition in this emerging research area; however, Khokhlov never followed on that first paper, while Warner and his various colleagues ran away with it.

Since the mid-1990s, the field has exploded, with dozens of original studies exploring all aspects of LCE behavior, often challenging the established knowledge in materials physics and applied mechanics. The year 1994 was especially pivotal: Finkelmann has established the method of two-step cross-linking to produce a monodomain LCE,^19,20^ the “Trace formula” was discovered to theoretically describe the equilibrium physical properties of nematic LCE, including their actuation and their “soft elasticity”,^21,22^ and also the essential mechanics of smectic LCEs were described, accounting for the coupling of network cross-links and smectic layers.^23,24^ All of these concepts are still in active use today. There are several extended reviews of that foundation period of LCE development, with most of the emerging LCE knowledge summarized in a key monograph.^7^ This Review tries to capture the main developments in this field in the years that followed the state of the art captured in that book and thus are not represented there.

The subsequent sections of this Review represent the key directions of this development: first in materials chemistry, where two cardinal breakthroughs have been achieved in the recent decade. Then we examine the modern approach and practical utilization of reversible LCE actuation, which has mainly occurred via 3D printing of aligned LCE filaments. Finally, we explore the anomalous dynamic-mechanical response of LCE, which leads to the enhanced damping and the associated pressure-sensitive adhesion, both highlighting at least one remaining fundamental unknown in understanding the LCE physics. Note that the ideas of “soft elasticity” are only briefly discussed in this Review: this remarkable property of LCEs is strictly the equilibrium phenomenon, and the only new development involving soft elasticity over these years has been in better understanding of the polydomain–monodomain transition in nematic LCEs,^25^ although even there the role of slow relaxation dynamics is dominant and never captured in equilibrium theories. We also will miss on the discussion of smectic LCEs.^23,26^ This is a rich subject with many unique and remarkable properties,^27−29^ demanding novel modeling approaches.^24,30,31^ However, there has been less progress here in the last 10–15 years, and this Review attempts to focus on the few recent breakthroughs in the LCE field.

Another aspect of this field that is *not* represented in this Review is the development and properties of densely cross-linked liquid crystalline networks (LCNs): in the dawn of this whole area of research, it was collectively decided to distinguish the two kinds of materials by using the LCE and LCN terms, respectively. Although both are the cross-linked networks of the same or similar rod-like mesogens, in the elastomeric LCE the longer chains between cross-linking points enable sufficient freedom of movement for the mesogens to orient themselves and retain their LC properties coupled to but evolving independently from the underlying rubbery network. In the densely cross-linked LCN (with just one mesogen between cross-links), the freedom for rotational motion is insufficient even above the glass transition, and the mesogen alignment is not an independently established thermodynamic LC phase but instead a mechanical consequence of surface alignment and/or network topology. The field of LCN is also rapidly growing, with many attractive applications,^32−34^ but here we stay firmly with the elastomeric LCEs, which are the blend of two worlds: combining the softness and high ductility of elastomers with the directional anisotropy of liquid crystals, resulting in several unique physical properties.

## Liquid Crystalline Elastomer Materials

LCEs are elastomer networks that are thermosets in which molecular components capable of mesogenic ordering are incorporated. As the molecules are tethered into a polymer matrix and no longer free-flowing as in classical liquid crystals, sufficient network flexibility is key to allow the development of the proper LC phase. To this effect, flexible spacers are used alongside the rigid mesogens to compensate for their confinement in the network and provide the required molecular mobility. The nature and length of the spacer have a strong impact on the overall properties of the LCE. In the early side-chain LCE, it was established that shorter spacers connecting to the backbone, especially with an odd number of carbons to induce a natural oblique angle of the mesogen axis to the backbone, promote the nematic phase with the prolate backbone anisotropy along the nematic alignment. Longer flexible spacers promote smectic phases where the backbone and spacers “microphase separate” from the mesogens, forming layers with oblate backbone anisotropy in layer planes.^26^ Additionally, the mere fraction of nonmesogenic spacers in the overall composition of elastomer is a significant factor. Increasing the spacer length and flexibility does add rotational freedom for the mesogens but also “dilutes” the rigid segments within the overall network.^35^ A too-high concentration of rigid rod-like segments will lead to insufficient flexible intermediary segments and thus restrict molecular self-organization and quench LC properties (as in LCN mentioned above). On the other hand, a too-dilute concentration will result in limited self-organization, as the mesogens are too distant to template their orientation off of each other.^36,37^

**Figure 2:**
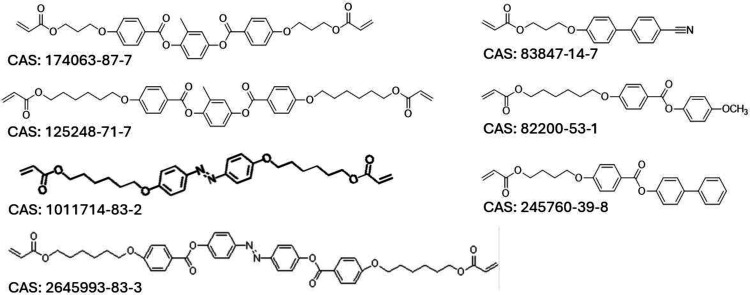
Examples of commercially available reacting monomers, with their CAS numbers for reference. The diacrylates are the mesogenic units used for main-chain LCEs, while monoacrylates form side-chain LCE materials.

The radical change in the state of the art has occurred with commercially available mesogens (called reactive mesogens, or RM),^38^ removing the need for multistep synthetic efforts required prior to this to obtain fundamental starting materials. Initially imagined by Broer in the late 1980s^32,39^ and commercialized by Merck in the 1990s, the compounds of the RM family have today become ubiquitous in the field of LCEs, supplied by many providers (see Figure 2 for several common examples of such reactive mesogens available commercially).

**Figure 3:**
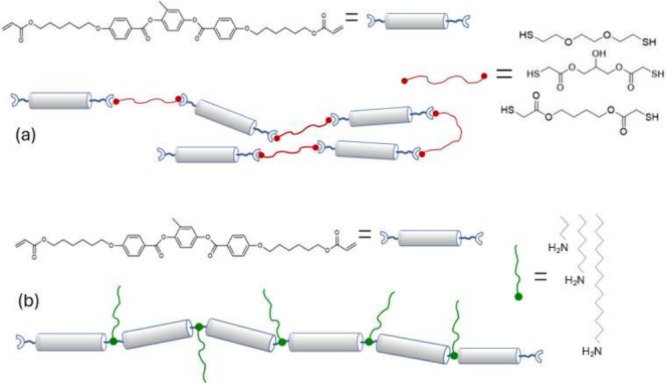
Two main topologies of main-chain LCEs in practical use today, utilizing the commercial RM monomers. (a) The main thiol–acrylate liquid crystalline main-chain topology, with a set of different possible dithiol spacers. (b) The amine–acrylate main-chain topology, with several amine monomers that could be used to link acrylate mesogens into a chain.

The breakthrough from the chemistry standpoint (following Finkelmann’s original development), facilitated by the ready availability of the RM series diacrylate mesogens, has been the introduction of thiol–ene “click” chemistry^40^ to the field for the synthesis of LCE networks.^41,42^ The removal of the need for starting monomer synthesis, combined with the ease and robustness of “click” reactions, made the field accessible to many nonchemist researchers in adjacent areas of research. The increased overlap with other areas of expertise marked a shift in focus from purely academic pursuits toward applications of LCE materials in different scenarios. Figure 3(a) illustrates the mainstream topology of alternating mesogenic units and a variety of flexible spacers in the main chain.^41,43^ The other popular construction of the main-chain liquid crystalline polymer is the amine–acrylate chain as illustrated in Figure 3(b), with several different primary amine linkers.^44,45^

**Figure 4:**
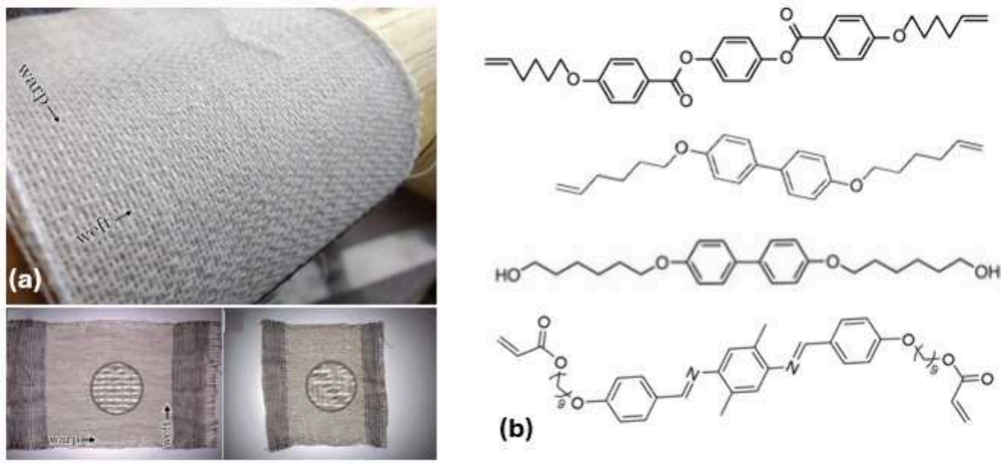
(a) Images illustrating the examples of woven fabric incorporating the strong LCE yarn. (b) Noncommercially available reacting mesogens used in the synthesis of main-chain LCEs, offering a different thiol–ene chemistry with divinyl mesogens, other synthetic routes with diol chemistry, and the diacrylate mesogen with exchangeable imine groups in the rigid core.

Other examples of polymerization reactions used to produce the liquid crystalline polymer chains and cross-link them into LCE networks include the epoxy–acid reaction,^46^ the epoxy–thiol reaction,^47^ the amine–acrylate reaction with hydrogen bonding,^48^ the thiol–isocyanate reaction,^49,50^ and of course the acrylate homopolymerization used as the cross-linking mechanism.^41,51^ As the mechanical properties and the phase transitions of an LCE material are dependent on its constituting chemistry, the development of a broad library of polymerization reaction candidates makes it possible to obtain elastomers ranging widely in material and mechanical properties, from a soft siloxane-based network to a leathery urethane-based system reinforced with hydrogen bonds and a stiff epoxy-based LCE system. A notable development in the pursuit of more stiff LCE materials, which would allow drawing strong LCE yarn and weaving the fabric, was the concept of a double network^52,53^ when two interpenetrating LCE networks are formed, where the resulting mechanical resistance to stretching is much enhanced. This has been already utilized in weaving LCE-containing fabric on a standard loom.^54^Figure 4(a) illustrates some of the active LCE textiles reported.

In terms of the base reacting monomers, although the field currently is dominated by the acrylate reacting mesogens (RM) mentioned above (Figure 2), the search for different variations continues. One of the other ideas of Broer in the 1990s was the divinyl reacting mesogen,^55^ which offers different reaction conditions from acrylates and is occasionally used today^56,57^ (see Figure 4(b)). One has to note that the same work by Broer^55^ also offered the thiol-terminated mesogen concept, which would give even more rich “click” chemistry options in LCE synthesis. In the similar vein, the diol reacting mesogens have been successfully used in the work of Zentel, which was the precursor to the modern 3D printing of LCEs.^58^ A recent example of a different mesogen concept is also shown in Figure 4(b): the imine-based rod-like mesogen is capable of bond exchange within the rigid rod itself, leading to alternative ways of forming vitrimer LCE networks.^59^ No doubt, as soon as some of these alternative mesogens show commercial promise, their bulk synthesis would also make them available from supplier catalogues.

In the next section, we will discuss one of the two main physical properties of LCEs leading to potential applications: reversible large-stroke actuation. However, ever since the first “single-crystal LCE” of Finkelmann,^19^ actuation has relied on the permanent orientational alignment in the LCE network, and that has been hard to achieve except in a few limited cases such as surface alignment in thin films or flow alignment in thin 3D-printed filaments. The difficulty of creating alignment patterns in a bulk material is the main dilemma in this field. To address it, a conceptual breakthrough has been the introduction of covalent adaptable networks,^60,61^ which today are often referred to as “vitrimers”, to the field of LCEs.^46^ Covalently cross-linked polymer networks (nominally, thermosets) could undergo the elastic–plastic transition at an elevated temperature (above the “vitrification” temperature *T*_v_) where the bond-exchange reaction rate becomes significant. Through this added network malleability, the door was opened to postpolymerization processing of the nominally thermoset materials, expanding the range of alignment patterns and the resulting actuating structures accessible, and pushing further out the limits of possibilities in terms of processing and applications. Today, there are at least a dozen different bond-exchange mechanisms tested in the LCE context, making what has been named exchangeable LCE (or xLCE), all with different positive and negative factors, but all aiming to achieve full reprocessability and realignment of LCE materials.^62^

In particular, the incorporation of vitrimer chemistry into LCE networks offers the possibility of material realignment by applying a required stress pattern at a temperature above *T*_v_. As the network chains are stretched out of their equilibrium coil configurations, the local network anisotropy is established via the exchange-mediated network rearrangements. Once the temperature is brought below *T*_v_, the exchange stops and the polymer network conserves the newly established local anisotropy pattern and, with it, the aligned state of the nematic order. Heating the samples again above the vitrification temperature *T*_v_ and applying another stress pattern enable full reprogramming of the alignment and the overall material shape, making xLCE renewable and recyclable. The added vitrimer properties include weldability and self-healing. Today, there are at least a dozen different bond-exchange mechanisms tested in the LCE context, all with different positive and negative factors, but all aiming to achieve full reprocessability and realignment of LCE materials. The recent review^62^ presents a detailed state of the art in the xLCE field, including the long list of different bond-exchange chemistries available.

**Figure 5:**
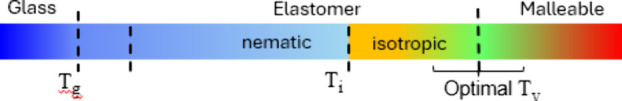
Schematic representation of the required xLCE characteristic temperature balance.

The temperature range for material malleability is of particular importance in the xLCE intended for actuation: here, three characteristic temperature thresholds need to be adjusted with care: the glass transition *T*_g_, the nematic–isotropic transition *T*_i_, and the vitrification point *T*_v_ (Figure 5). *T*_g_ must be below room temperature to maintain the elastomeric nature of the ambient material. *T*_v_ defines the start of the material malleability. If *T*_i_ were close to or above *T*_v_, actuation would be impossible: the network would be in the plastic-flow regime and could not retain shape. Hence, for actuation, the condition *T*_i_ < *T*_v_ must be respected, with a sufficient gap such that when temperatures are increased above *T*_i_ a gradual creep due to a slow onset of material malleability does not deform the material over the course of multiple actuation cycles. A minimum of 30 °C for this gap has been postulated as a safe value. Finally, the elastic plastic transition must occur below the thermal degradation of the polymer. All of these factors limit the choice of bond-exchange chemistry, forcing the rate of this exchange into a narrow optimal range.

The current surge of activity in the field of exchangeable LCEs generates an expanding toolbox for malleable systems, from different methods to incorporate a type of exchange reaction within a given network chemistry to allowing new exchange dynamics and kinetics and expanding the temperature range accessible for material malleability.

## Liquid Crystalline Elastomer Actuation

**Figure 6:**
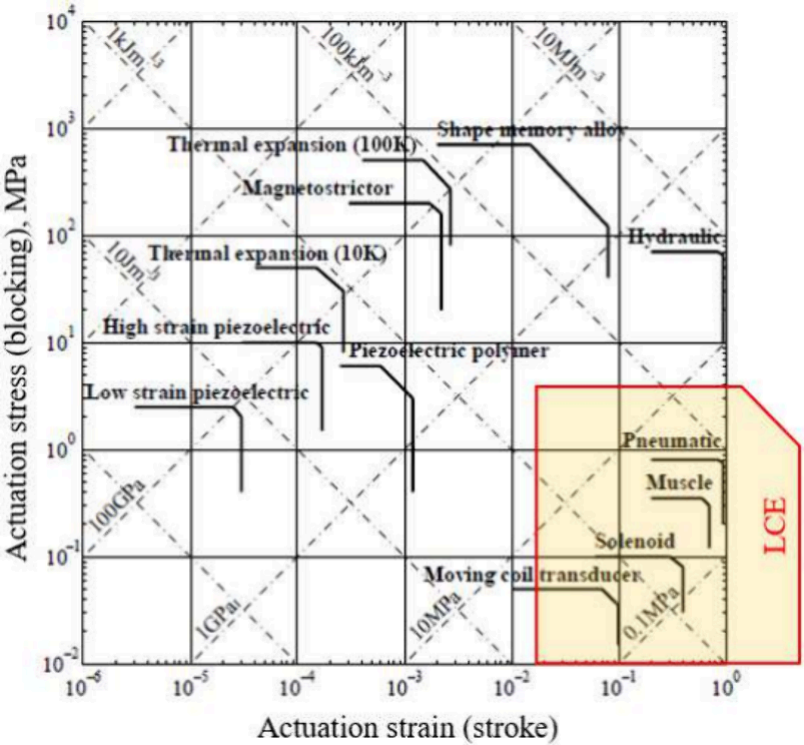
“Map” of actuators, indicating their stress and strain range. LCEs demonstrate properties in a similar range to those of muscles but are in principle capable of much greater actuation stress and strain. Figure concept is adapted from ref^65^.

Their inherent softness (above the glass transition) stemming from their entropy-dominated rubbery nature, combined with their tuneability through their rich chemical composition, makes LCE actuators competitive materials in a number of fields, such as robotics^63^ and biomedical engineering.^64^ It is instructive to examine the place LCE actuators would occupy on the general “map” of actuating systems presented in a key mechanical engineering review recording the state of the art in 1997^65^ (see Figure 6). The record actuation stroke in LCE has been reported to be around 300–500%,^66,67^ although most practically relevant LCE systems operate in the stroke range of 20–50%. Being rubbery, LCE are unlikely to offer blocking stress exceeding their base rubber modulus (within the range of single MPa), although the mentioned developments of double networks may push this boundary further up.

Initially theorized as an artificial equivalent of choice to mimic muscle tissue,^16^ the scope of interest and applications of LCE materials has greatly expanded, with demonstrated results in areas such as sensors,^68^ modulated surface coatings,^69,70^ tissue engineering,^71,72^ and the already mentioned active textiles,^54,73^ among others. A state of the art review of this area is available for a deeper literature exploration.^74^

The natural state of a LCE material formed without any constraint is the polydomain state (akin to the polycrystalline state in hard solid matter), where its constituting mesogens self-organize into microdomains of nematic alignment of approximately 1–2 μm in size;^75,76^ each microdomain orientation is independent from its neighboring microdomains, resulting in a patchwork of microscopic randomly aligned regions (although a remarkable study by Uchida^77^ has suggested that local elastic compatibility could force a certain coherence in the domain alignment pattern). The overall material is hence isotropic, when averaged over many domains, but has a very high light scattering due to the randomly aligned birefringent domains and is optically opaque.

When aligned, either during polymerization in an external field or due to the mechanical constraint of a polydomain material (ultimately, a pattern of local uniaxial stretching), the LCE material transitions into a monodomain state: in this state, all the microdomains align to generate a macroscopic region with a single global director ***n*** for the material. The material in this state is transparent. In an aligned state, the mesogenic orientation causes a distortion of the standard spherical coil configuration of the polymer chains, with an average prolate (or oblate) shape for the chains becoming more energetically favorable as a compromise between chain coil configuration and optimizing LC ordering, as illustrated in Figure 1(c). In the monodomain state, this local chain anisotropy translates into the whole body shape of the LCE.

If the material is polymerized so that the mesogens are aligned within the network in its state of equilibrium, then actuation becomes possible. The change in the mechanical shape of the body occurs spontaneously when the system transitions from an uniaxially ordered state to an isotropic state, so that average prolate (or oblate) anisotropic network strands turn become spherical. This transition most commonly occurs when the LCE is heated above the isotropic transition temperature *T*_i_ (there are many ways to inflict heating onto an elastomer). Extensive literature covers this phenomenon, unique to LCEs.^7^ Less common (but no less famous) is the actuation due to photoisomerization, when the transition temperature is made to shift below the constant operating temperature.^78,79^ Studies refer to “photo-actuation” when a light stimulus is used to trigger the change in local order, and the associated with it change in sample natural shape. The physical origin of this effect could be due to azobenzene isomerization, for example, but in many cases the underlying effect is still the local heat produced by light absorption in dyes^80,81^ or nanoparticles.^82,83^ Electric or magnetic triggering of actuation is, of course, much more desirable from the technology point of view, and examples of this LCE actuation all involve dispersing nanoparticles, or even liquid metal, in the elastomer matrix.^84−86^

The original interest in LCE actuation stemmed from the pioneering uniform alignment of Finkelmann’s thin films formed via the two-step cross-linking method.^19,66,87,88^ Ultimately, all modern successful methods of LCE alignment are based on this two-step cross-linking concept. The only exceptions are the surface-induced alignment of thin films^32,42^ and the cross-linking in external electric/magnetic fields,^89,90^ and each is only applicable in a specific narrow window of conditions and limited LCE dimensions (e.g., the thickness of the LCE for this alignment to work can never be greater than ca. 100 μm). Recent review articles thoroughly cover this aspect of LCE materials.^91,92^ Again, there are a few exceptions when a thicker LCE film was aligned by a strong magnetic field (up to 1.25 mm)^93,94^

**Figure 7:**
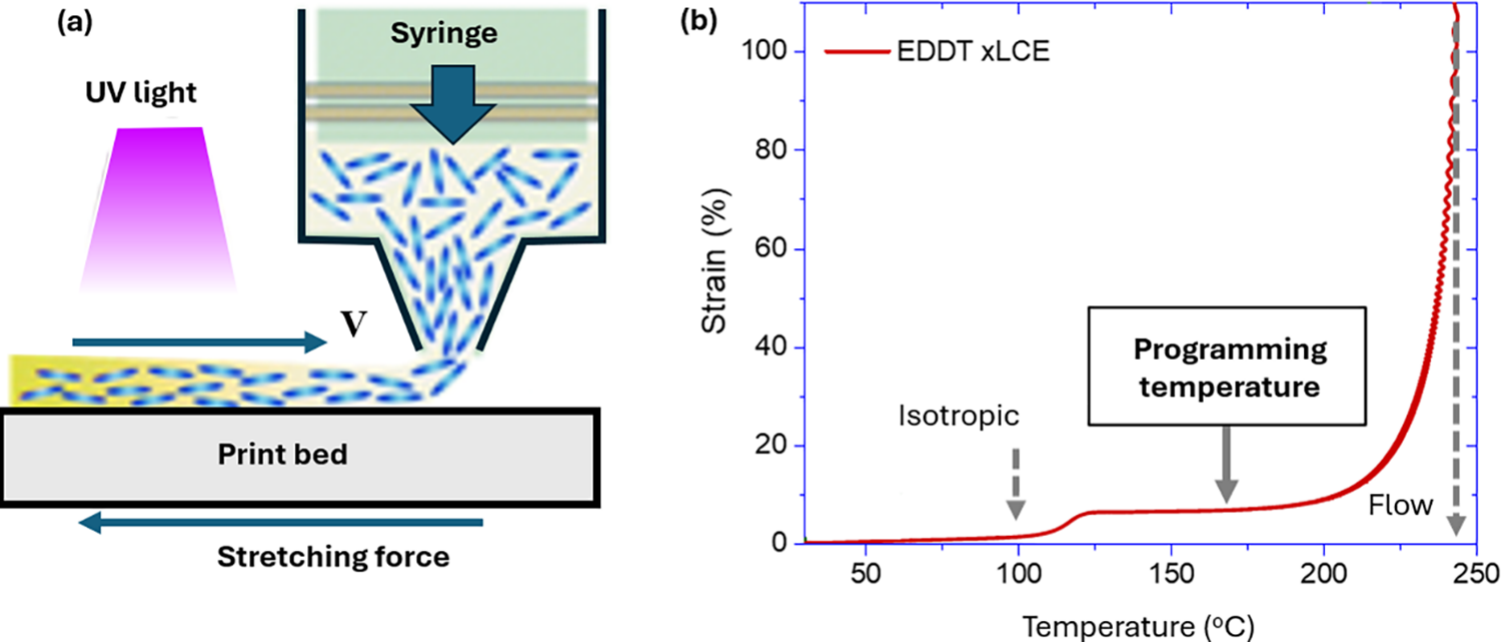
(a) Schematic representation of LCE alignment during DIW extrusion of the polymer mix (figure concept adapted from ref (95)). (b) Creating and fixing the alignment of a large-scale LCE sample requires the exchangeable bonds and elastic–plastic transition. The plot shows how one finds the optimal temperature for such programming: in the isotropic phase but below the vitrification point *T*_v_, so there is slow plastic creep under applied stress.

Currently, most of the focus in LCE actuators has shifted into one of two directions: 3D-printed LCE filaments, often forming intricate constructions, and large-scale bulk LCE structures capable of delivering mechanical work. In the first case, the alignment method is effectively the two-step crosslinking, when the shear-aligned precursor extruded from the nozzle is stretched and cross-linked, usually by UV utilizing either acrylate homopolymerization of thiol-vinyl bonding, see the sketch in Figure 7(a). Creating and fixing the alignment of a large-scale LCE sample requires exchangeable bonds and an elastic-plastic transition. The plot in Figure 7(b) shows how one finds the optimal temperature for such programming: in the isotropic phase but below the vitrification point *T*_v_, so the slow plastic creep under applied stress could be controlled.^96^ It was postulated that when such creep reaches about 100% strain, the temperature could be lowered and the anisotropy would remain permanently recorded in the network, causing the nematic order to form uniformly in that direction when *T* < *T*_i_.^97^

The material formation in the 3D printing method relies on the mesogenic oligomer chains formed prior to printing (as in Figure 3), constituting the “ink” used for printing; the ink is extruded through the nozzle to “write” in the form of a polymer bead naturally aligned on extrusion on the substrate. The cross-linking is triggered immediately on leaving the nozzle to fix the state of filament alignment. Intricate local alignment becomes accessible through this technique,^98,99^ as well as complex architectures.^100,101^ However, an inherent limitation of this technique is the impossibility for out-of-plane alignment, as alignment is restricted to the printing path. Other 3D printing methods that have been used for the additive manufacturing of LCE materials include direct laser writing by two-photon polymerization, digital light processing, inkjet printing, and fused deposition modeling, all possessing the same shortcoming. However, in spite of any shortcomings, the 3D printing of LCE materials holds great potential and is a very active area of research.^95,102^

**Figure 8:**
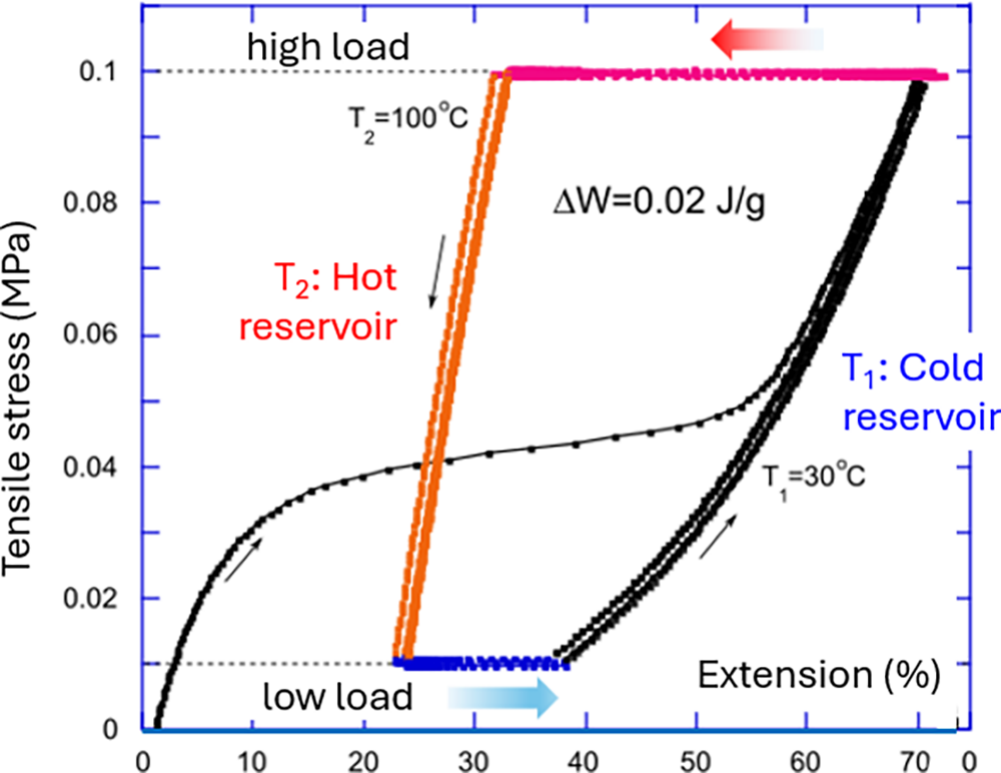
Stress–strain plot of several consecutive “heat engine” cycles following the initial loading curve starting from (0,0). After that, the four steps of the repeated cycle are as follows: (1) heating at constant high load (the power stroke), (2) unloading while in the hot state (*T*_2_), (3) cooling at constant low load (recovery stroke), and (4) loading, while in the cold state (*T*_1_). The plot has 11 cycles that overlap almost perfectly after the initial settling-in.

Bulk LCE actuators are designed to generate a meaningful force and output mechanical work during the actuation cycle, something that is not possible in thin-film or filament systems designed for bending on actuation. There are not many practical examples of this in the literature, one of them being the large-scale heliotracking platform.^96^ It is instructive to assess the limit of such mechanical work output, in a set of multiple typical “heat engine” cycles illustrated in Figure 8. Here, the polydomain LCE actuator is stretched at “low temperature”, initially tracing the familiar stress–strain curve of the polydomain–monodomain transition reported in many LCE studies.^7^ Then the sample is kept at constant load (of 0.1 MPa in the plot), aligned, and heated to a “high temperature” (above *T*_i_), which causes it to contract: this is the “power stroke” of the engine. Then the sample is unloaded (to 0.01 MPa in the plot: the stress below the level of soft plateau) while keeping the temperature constant, reaching the shortest length. After this, the sample is kept at constant (low) load and cooled to low temperature, where it spontaneously elongates due to the remaining nematic alignment that is not given time to fully return to the polydomain state. Then it is stretched (loaded) again, while at low temperature, and the cycle continues. The plot in Figure 8 shows that after the initial loading step, the heat/load cycles are perfectly repeatable, with the shape of the cycle depending on the rate of temperature/load changes applied. As in basic thermodynamics, the area of the cycle represents the useful work output (or the work input if the clockwise cycle is employed instead to represent the heat pump).

In the counterclockwise cycle of Figure 8, the heat engine outputs the mechanical work or ca. 0.02 J/g on each cycle. Is this a lot, or just a small fraction of what the LCE is capable of? One could think that the absolute maximum of internal energy involved in actuation cycle, which is driven by the phase nematic–isotropic transformation of the LCE, is the latent heat (enthalpy) of this transition, which one could often determine by differential calorimetry. The nematic–isotropic transition is the “weak first-order” transition,^103^ and although there is great variation of transition enthalpy in LCEs depending on their composition, in this author’s experience the maximum transition enthalpy is seldom above Δ*H* = 0.2 J/g. This should be the limit of useful work that is possible in a bulk LCE heat engine.

The response time of actuation is a very significant parameter that is often hard to assess. Studies have shown that the equilibrium LCE response of adjusting the shape to the current temperature is limited by the rate of heat transfer in bulk materials.^104^ This makes the bulk LCE actuators inevitably “slow”, while thin films and filaments are able to respond faster. The internal rubber relaxation rate (related to the Rouse time of the network strands) is much faster, a fraction of milliseconds, and it is unlikely that any LCE device would explore this limit of response rates. All of these dynamic factors should be considered when designing LCE materials for actuation, depending on the desired outcome and application.

## Soft Elasticity and Polydomain State

**Figure 9:**
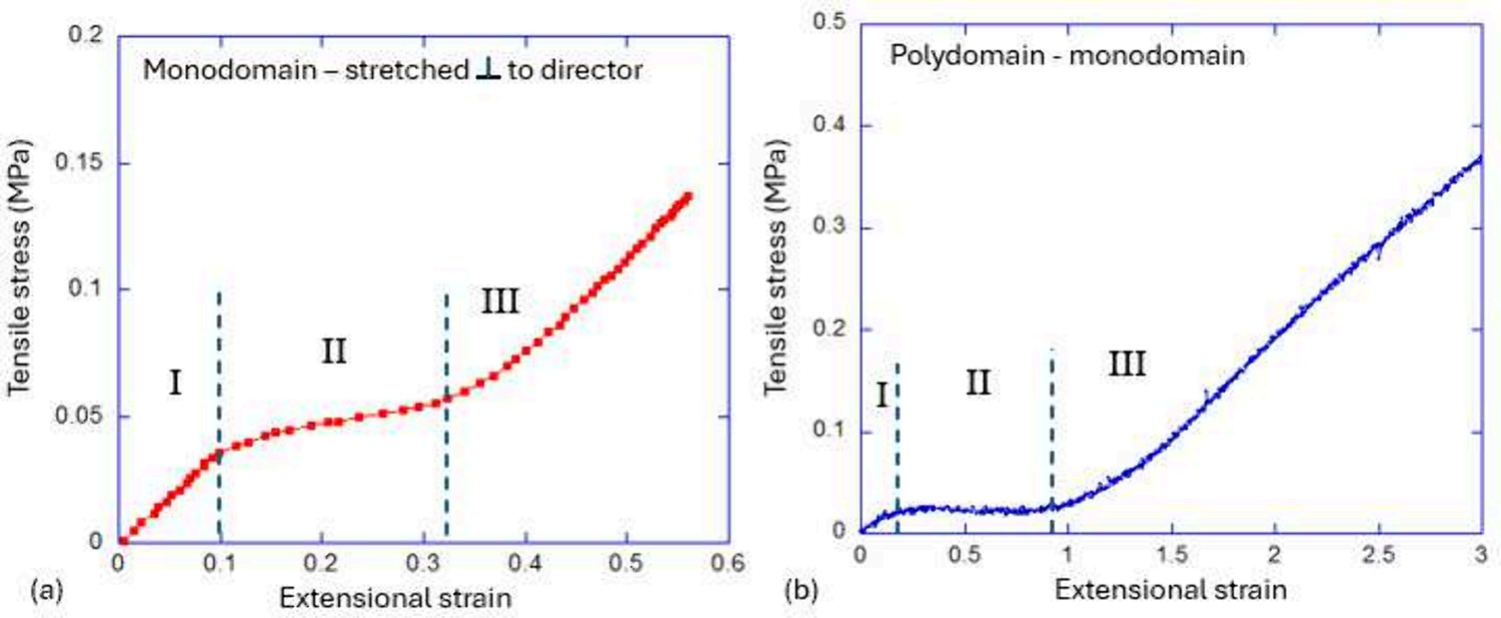
(a) The stress plateau observed during the soft director reorientation of a uniform LCE stretched perpendicular to its initial director (data from ref (108)). (b) The stress plateau observed during the PM transition on stretching a polydomain LCE. Both plots mark the regions of the initial semisoft rise (I), the stress plateau where the director rotation occurs (II), and the final region of stretching long the fully aligned director (III). The materials and settings are very different in these studies: plot (a) is for a side-chain LCE aligned perpendicular to the film plane, while plot (b) is for the main-chain thiol–acrylate polydomain LCE.

The phenomenon of soft elasticity was understood in the early days of LCE research. Theoretically, it follows directly from the “Trace formula” describing the elasticity and its coupling to director orientations^22^ or from the phenomenological symmetry arguments.^105^ Experimentally, it has manifested in many settings, such as the stripe domains that develop in the uniform LCE stretched perpendicular to its director^106^ or in the reduction of the linear shear modulus when the geometry of deformation induces director rotations.^107^ The key mechanical characteristic of soft elasticity is the stress plateau extending over the whole region where the director rotation occurs without resistance, as reported in many studies^7,108^ (see Figure 9(a)).

Equally, the fact that an LCE network always forms a polydomain structure in equilibrium (and returns to it after annealing in the isotropic phase, which is a great contrast with ordinary liquid crystalline textures where the true equilibrium is the state of uniformly aligned director) has been known for a long time. The first mechanical trace of the “polydomain–monodomain” (PM) transition was reported by Schätzle and Finkelmann in 1989,^109^ and the first theoretical understanding of the origin of equilibrium polydomain state and the nature PM transition emerged about a decade later.^76^ It has become clear that the pronounced stress plateau, as seen in Figure 9(b), observed on uniaxial stretching during the whole range of domains rotating from their random initial orientation to the uniform alignment forced by the mechanical force, also has to do with soft elasticity, only at the level of microscopic domains.

In both cases, one is aware of the nonzero stress on the soft plateau (II) and a small linear stress–strain region before its onset (I). Again, much has been written about “semisoft elasticity” over the years,^7,110^ and to briefly summarize it here: the stress plateau would be zero in an ideal LCE with no internal constraints. Semisoftness is called that because the symmetry of deformation is exactly that of the soft pathway, but internal constraints create a barrier for its onset. There are different physical origins of semisoftness in a uniform monodomain LCE, varying from microscopic compositional fluctuations to chain entanglements in the cross-linked network and macroscopic mechanical constraints applied to the body. In all cases, the effect could be expressed as a factor added to the ideal “Trace formula” for the elastic energy density:
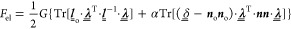
1where *G* is the rubber modulus,  are the chain anisotropy tensors before and after deformation (uniaxial along the nematic director ***n***), and  is the deformation tensor. All the details of the Trace formula and its implications can be found in ref (7). Importantly, the parameter α tells the relative strength of these internal constraints and determines the level of nonzero stress plateau. Semisoft response arises because of the symmetry mismatch between the two terms in Equation 1: the geometry of soft deformation when , with  as the arbitrary rotation matrix, does not have the same effect on the tensor combination in the added semisoft term, and the elastic response is nonzero, although still much lower if α is small. This logic explains the observation of deformations accompanied by the director rotation in a uniform monodomain LCE, as in Figure 9(a).

**Figure 10:**
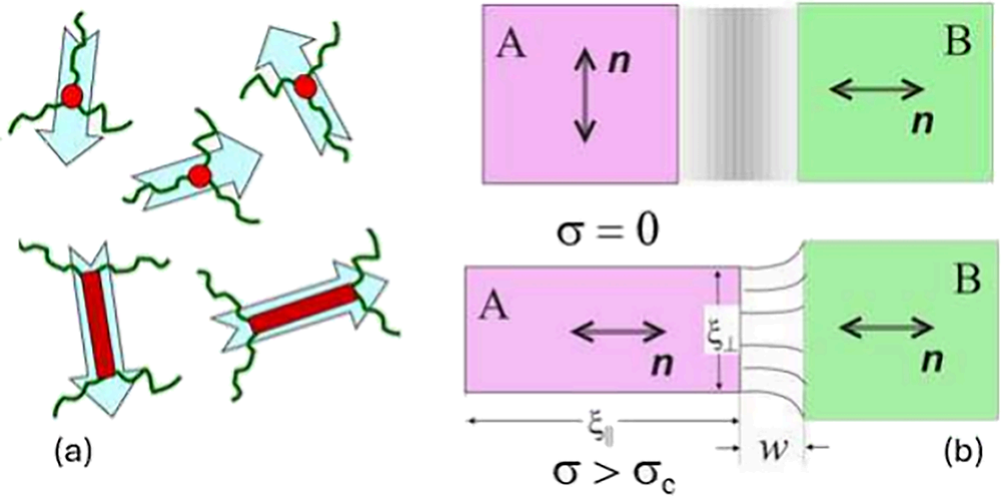
(a) Sketch illustrating local anisotropy introduced by various network cross-links, randomly placed in an isotropic-genesis LCE. (b) The sketch illustrating two neighboring domains, one of which (A) is forced to rotate its director by the applied uniaxial deformation (horizontal in the picture). The localization of domain wall *w* is explained in the text.

To understand what happens during the PM transition in the stretched polydomain LCE, one needs to recall the origin of the polydomain state in equilibrium.^76^ Random anisotropic cross-links in the network have much reduced mobility compared to the mesogenic chain segments, cf. Figure 10(a). This causes the effect of “quenched random disorder” and produces the equilibrium director pattern similar to spin glasses: large regions (domains, of the size 1–2 μm in practice^75^) are able to align in spite of the many point sources of random anisotropy, but this locally uniform director alignment cannot be sustained on a larger scale (the penalty for mismatch with the random anisotropy sources becomes too high) and the correlation of the director orientation is gradually lost over the distance (ξ, 1–2 μm in practice) that we call the “domain size”, although there is no sharply defined domain boundary.

**Figure 11:**
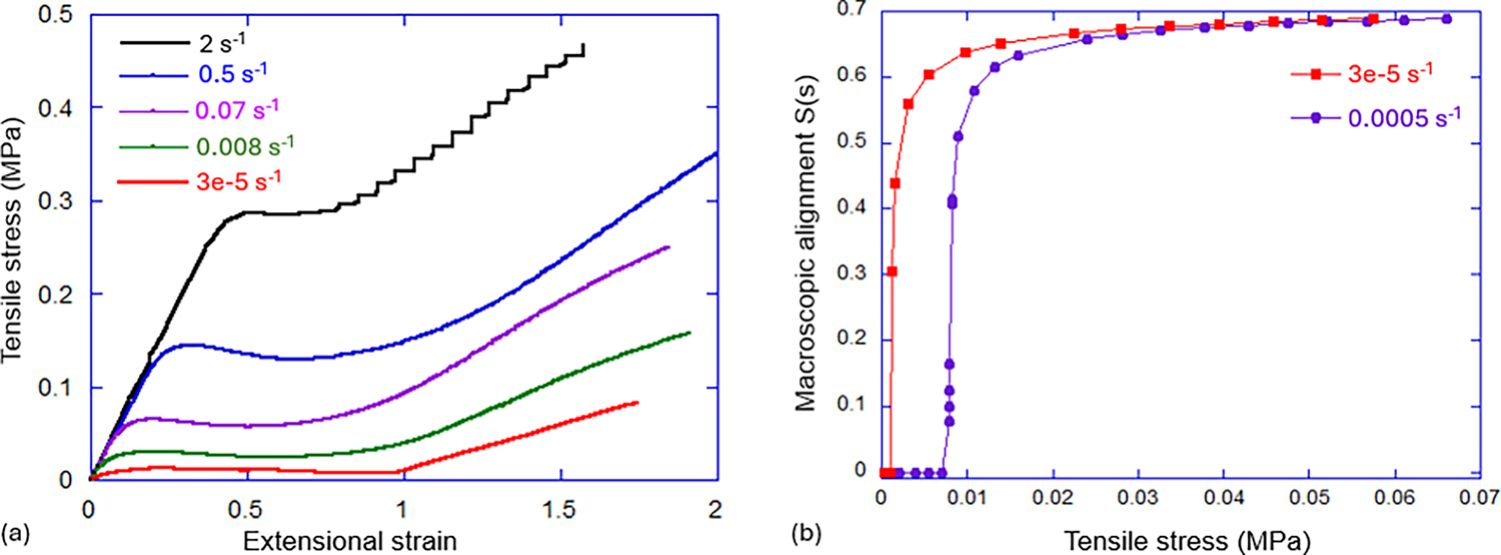
(a) The stress plateau observed during the PM transition in a main-chain thiol–acrylate LCE. Different curves show the trace at different rates of applied strain (labeled in the plot). (b) The macroscopic alignment, measured from X-ray scattering at different points during deformation, shows the evolution of *S*(σ) at two different rates of deformation. Data are from the study of stress-induced polydomain LCE alignment.^112^

When a uniaxial tensile stress is applied, each “domain region” attempts to rotate its director toward the stretching axis; some have greater angles to rotate than the others, as shown in the sketch in Figure 10(b). In an incompressible elastomer, this causes an elastic mismatch in the boundary region between such different domains; in the sketch, domain A elongates to ξ_∥_ and becomes thinner to ξ_⊥_ in the transverse direction. The interface between such mismatched domains becomes sharper, and it has been estimated to reduce as
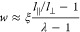
2where the key parameters are the nematic chain anisotropy ratio _∥_/_⊥_ and the imposed strain λ – 1. This estimate of domain wall localization and the elastic energy contained within it allows the estimate of the threshold stress for the PM transition (and given that above this threshold the stress plateau is flat, the level of the stress plateau itself): σ_c_ ≈ *G*(_∥_/_⊥_ – 1) in equilibrium. In practice, the slow internal relaxation in a LCE makes the equilibrium very hard to achieve and the plateau stress level strongly dependent on the rate of deformation. Figure 11(a) illustrates these characteristic PM transition stress–strain curves at different rates of deformation; one might speculate that in a true equilibrium, the stress plateau is very low indeed.

Similarly, the degree of macroscopic alignment, which gradually increases as the LCE is stretched along the stress plateau, has been estimated to depend on the applied stress:
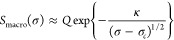
3where *Q* is the underlying local temperature-dependent nematic order parameter (which, in a polydomain system, could only be determined by sophisticated techniques such as dNMR^111^) and the constant κ is the result of the quenched disorder analysis of this problem.^76^

However, in the polydomain LCE the internal constraints are much more complicated than suggested in the estimates above: the need for neighboring domains to mechanically comply with each other when they rotate their director in a different way makes this a complex mathematical problem. The significant development in the last two decades has led to a much better understanding of what actually happens in this process of forced reorientation of microscopic domains.^25,77,113^ Based on the quasi-convexification theory of Conti and DeSimone,^114^ the modern constitutive relation describing the PM transition has been derived in both the ideal and the semisoft elastic case of the underlying LCE matrix. Today, one can computationally predict the complex stress and orientation patterns formed in polydomain LCE under much more elaborate deformations than a simple uniaxial stretch. Examples of Hertzian contact (when a spherical solid particle is indented into an LCE layer) and “anti-Hertzian” deformation (when LCE surface is compressed with a round hole, forcing the material to form a spherical bulge into the opening) illustrate the power of such computational methods of LCE analysis.^112,115^

## Damping and Adhesion

It may appear to the reader that the LCE field as a whole is generally “well understood”, and the only challenges remain in designing new ever-elaborate materials and the search for better applications. In many aspects, this is a true state of the art reflection; however, there remains a significant “blind spot”, the question where there are plentiful experimental observations and clear trends, but no good theoretical understanding. This refers to the general problem of LCE dynamics and specifically the anomalous mechanical damping in these materials.

The phenomenon was first formally reported in 2001,^116^ where an anomalously high loss factor was found across the whole range of the nematic LCE phase. In the linear dynamic-mechanical study, the loss factor is defined as the ratio of imaginary and real parts of the complex linear modulus for oscillating deformation, tan δ = *G*″/*G*′, and is a function of frequency and temperature. It is directly related to the “Q-factor” routinely used to characterize the vibration and resonances of structures, as well as other oscillating systems where one finds a phase-shifted response function, e.g., electromagnetic or dielectric^117^ (in all cases, tan δ = 1/*Q*). In mechanical oscillations, solids usually have a very low loss factor: usually below 0.1, for both crystalline and amorphous glass systems. Elastomers, such as natural rubber, polyurethane, silicone, etc., have loss factors in the range of 0.1–0.3, which is why rubbers are often used as dampers between solid structures. Between these regimes (solid and elastomeric), there is usually a glass transition (at *T* = *T*_g_) where tan δ has a pronounced rise, often reaching peak values of 1.5–2. There is a simple qualitative understanding of this well-known effect: in the middle of glass transition, the barriers for particles (e.g., chain monomers) to escape their confining “cages” become significant, while not yet prohibitively high as in the deep glassy state. Hence in the middle of glass transition the molecular motion still occurs, but with a significant energy expended to overcome these increasing “cage” barriers, which is where the mechanical energy gets lost, and the growth of loss factor reflects that. However, in LCEs, the findings are that tan δ could be as high as 1 (or even higher) across the whole range of nematic phase before finally dropping to the “normal” low levels in the isotropic phase of the elastomer. Obviously it is the nematic order that is responsible for this anomalously high dissipation loss.

**Figure 12:**
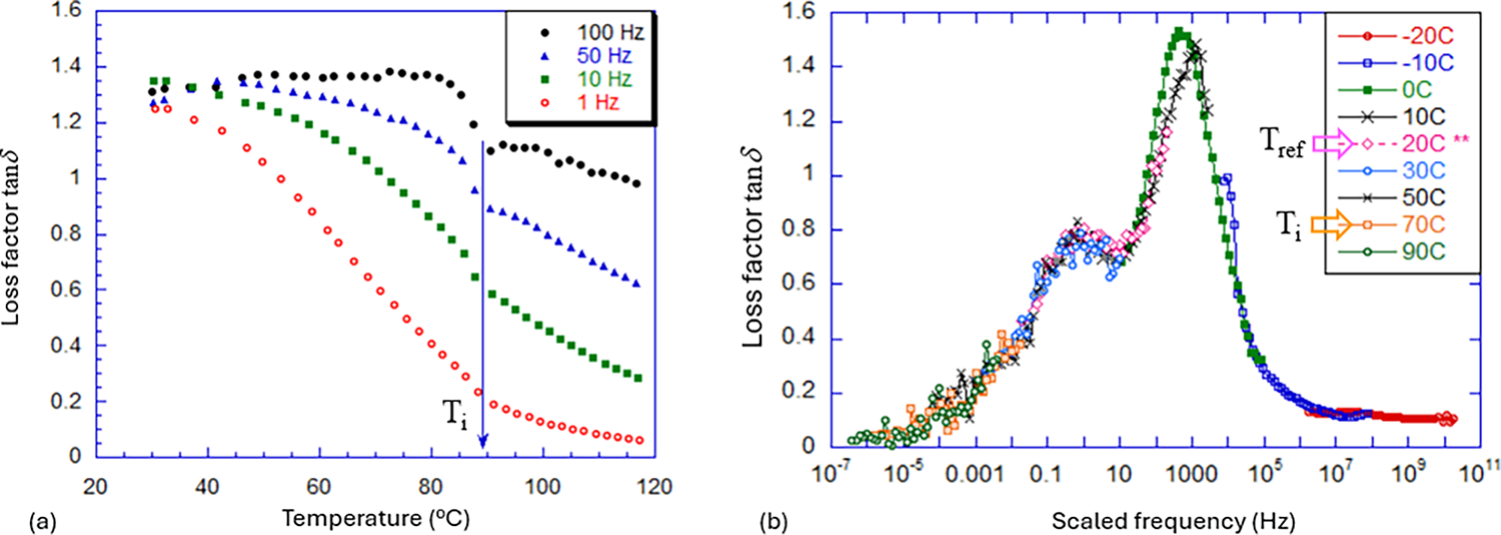
(a) The loss factor measured in a linear dynamic-mechanical test on side-chain polysiloxane LCE at several fixed frequencies (labeled in the plot). Data from ref (116). (b) The Master Curve for *T*_ref_ = 20 °C, constructed from linear dynamic-mechanical tests on main-chain thiol–acrylate LCE carried out at different temperatures (labeled in the plot). Data from ref (122).

There has been a long-established misconception in this field, introduced early in the 2000s and caused by the widespread fascination of soft elasticity. This author is certainly guilty of it, introducing the term “dynamic soft elasticity”.^107^ Indeed, it is true that in a dynamic (oscillating) experiment, one could impose a deformation geometry (shear) that causes director rotation. This will cause the decrease of the storage modulus *G*′ (just like we saw the decrease of the slope of stress–strain curves in soft elasticity), and as a result tan δ would increase. This could be called dynamic soft elasticity, but has become clear now that this has nothing to do with the observed anomalous damping observed. The main reason is to do with rates and frequencies: soft elasticity and any modulus reduction due to director rotation is the equilibrium effect. That is, the modulus would decrease more at lower frequencies because more time is given to stress relaxation on each cycle. Stress relaxation is notoriously slow in LCE,^118^ and to explore the modulus reduction described by the equilibrium theory, one needs superlow frequencies. Yet, even in the founding 2001 paper^116^ it was shown that the loss factor is higher at higher frequencies, e.g., reaching tan δ = 1.4 at 100 Hz (see Figure 12(a)).

It is not straightforward to construct Master Curves for the dynamic mechanical response in LCEs because the classical time–temperature superposition^119,120^ is modified by the changing nematic order. However, over the years, researchers have learned how to extrapolate the time–temperature superposition into the nematic LCE and produce valid Master Curves,^107,121,122^ such as the one illustrated in Figure 12(b). The meaning of such a superposition of frequency scans made at different temperatures is to interpret the “scaled frequency” at a fixed reference temperature. Thus, the plot in Figure 12(b) shows how tan δ would behave if measured between 10^–7^ and 10^10^ Hz at a constant temperature *T*_ref_ = 20 °C. (In fact, it is impossible to span such a frequency range in any real experiment, which is why the time–temperature superposition was invented.)

**Figure 13:**
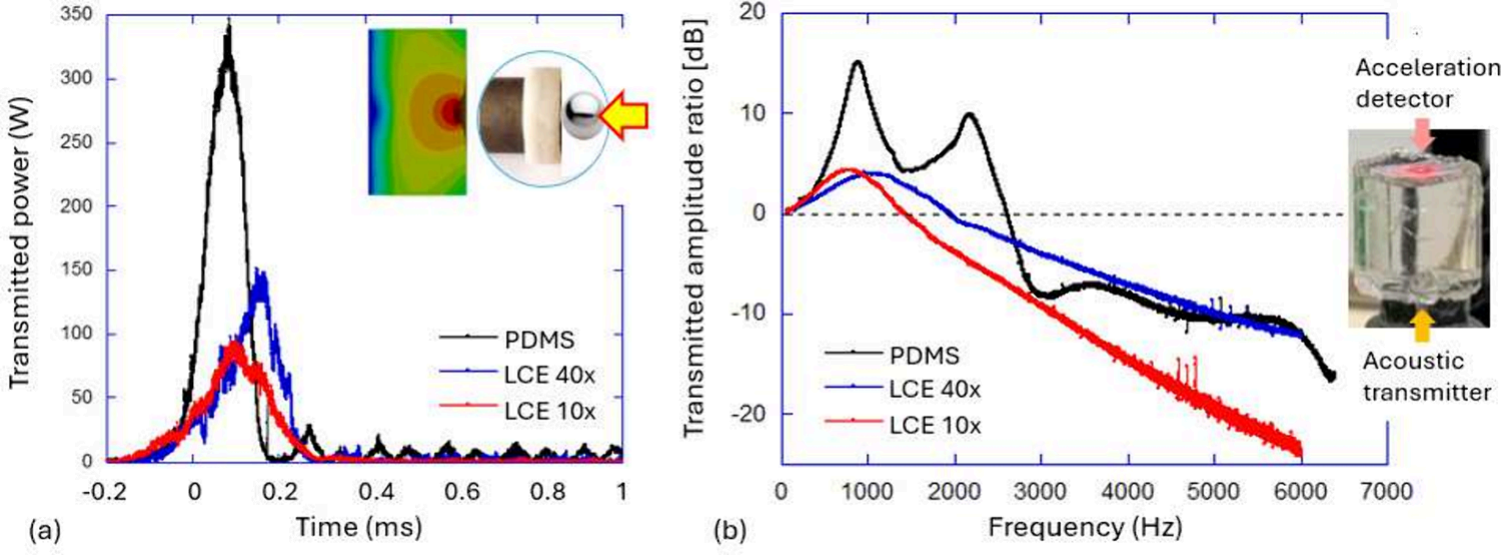
(a) The impact test of a spherical projectile on a flat damping pad. (b) The vibration attenuation test. In both tests, the damping pad made of soft PDMS elastomer is compared with the main-chain thiol–acrylate LCE with 40% and 10% cross-linking density, as labeled in the plots. All data from ref (122).

Whether we understand the underlying physics or not, the effect of anomalous damping of mechanical vibrations exists without any doubt, and there have been many recent studies aiming to exploit this in practical settings. The first aim has been to see if this damping translates into impact, where the sharp pulse of force applied to the LCE can be represented as a superposition of oscillations with a wide range of frequencies.^122,123^ The plot in Figure 13(a) shows the transmitted power after a spherical projectile hits a flat damping pad (as illustrated in the graphic), comparing the effect in two LCEs with different cross-linking density and a soft silicone elastomer. It is clear that the LCE exhibits better impact protection compared with a classical elastomer; especially visible is the lack of secondary oscillations from the acoustic wave bouncing in the pad. However, impact tests are sometimes ambiguous to interpret: what does one mean by “protection”? The impact energy dissipation in an LCE pad is very high, and the restitution coefficient in rebound from the LCE is very low; however, it is the maximum force delivered to the target that is of concern in impact. This maximum force is determined by the momentum transfer rather than energy dissipation,^124^ and LCEs with their slow response are not necessarily performing better in this respect.

The damping of vibrations in oscillating loading is a different mechanical scenario, probing dissipation at different fixed frequencies.^122,125^ Here the advantages of anomalous LCE damping are much more transparent. Figure 13(b) shows the results upon measuring the transmitted acoustic wave traveling across an elastomer pad (as illustrated in the graphic). As the transmitter delivers vibrations at controlled frequencies, the acoustic waves find characteristic resonances, determined by the geometry and the stiffness of the pad. For these resonance frequencies, the transmitted amplitude is higher than the input, and the logarithmic ratio plotted in the graph (expressed in dB) is positive. Again, the comparison between a silicone elastomer and two LCE variants is illustrated. The first resonance peak at 800–900 Hz is due to the longitudinal compression wave, which travels at approximately the same speed in all elastomers, given their similar densities and moduli. The subsequent resonances seen in PDMS involve shear wave components, and those are completely absent in LCE where shear waves have anomalous attenuation.^126^ It is also instructive to compare the resonance features of the one peak present in all of the materials. The Q-factor of this resonance for PDMS is *Q* = 2.4, which represents a decent attenuation in a soft rubber. In LCE samples, the observations are starkly different: *Q*_40x_ = 0.42 and *Q*_10x_ = 0.45, both representing an overdamped regime. It is unique for a thermoset elastomer material itself to be in the overdamped regime, but we already saw that tan δ = 1/*Q* can easily be in excess of 1 in the nematic LCE.

One remarkable property of high-damping polymeric materials is their pressure-sensitive adhesion. Anecdotally, everyone knows that soft rubbers feel sticky. By introducing the idea of the “viscoelastic trumpet”, De Gennes^127^ started the process of increasing understanding of how the internal entropy of polymer segments in contact with a solid wall leads to what we perceive as physical adhesion, which is an energy barrier (and thus the force required in pulling) to separate such a polymer from the wall.^128−131^ With the anomalous viscoelastic damping in nematic LCE, one should expect a parallel phenomenon of enhanced physical adhesion, which is also reversible when the material goes into the isotropic phase and back into the LCE again. The first formal report of such an enhanced adhesion has confirmed such an expectation.^132^

In thinking about physical adhesion, one must quickly dismiss any ideas about the surface energy γ_0_ determined by chemical compositions of the elastomer and the substrate, which determines the wetting contact angle, for example. The highest surface energy is in the range of 0.05–0.1 N/m (72 mN/m for water and air, one of the higher surface tension surfaces). Yet, the work of adhesion of some popular pressure-sensitive tapes is many orders of magnitude higher: the 3 M Scotch tape has the work of adhesion γ = 250 N/m and the 3 M Duct tape has γ = 700 N/m. It is the internal viscoelasticity of the polymer that is responsible for this adhesion and not the chemical affinity.

**Figure 14:**
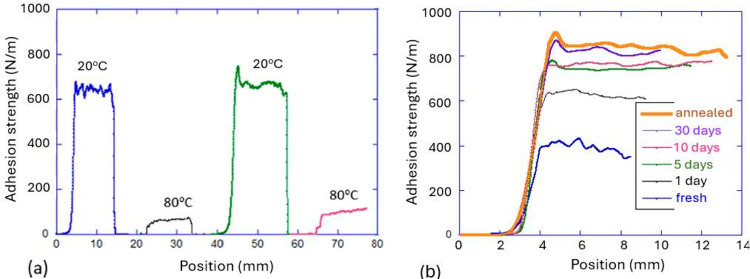
(a) The reversible strength of adhesion is illustrated in the peel test on the same sample, which was twice heated and cooled while attached to a glass plate. (b) The illustration of long relaxation and the effect of annealing to reconfigure nematic domains in contact with the surface: the peel test is carried out after different times of contact with substrate. In both plots, the adhesion strength γ (often called the work of adhesion) is the peel force per unit length of contact line. All data from ref (134).

There are three typical tests and settings where physical adhesion of two surfaces is probed: the lap shear (from the term “overlap”), the peel, and the probe tack. The latter two have been shown to probe essentially the same property, i.e., the “tackiness” of the surface, and their results are directly correlated.^133^ In contrast, in the lap shear geometry, the strength of the polymer plays a much greater role since the shear strain in the thin adhesive layer could reach very high values. These test conditions have been carefully compared in a recent study,^134^ confirming the reversible adhesion strength: high in the nematic phase and low in the isotropic phase (see Figure 14(a)). Here, a 5% cross-linked thiol–acrylate main-chain LCE with an isotropic transition *T*_i_ = 60 °C was used as a thin polydomain layer coating a PET backing tape, testing adhesion to glass and confirming a large and reversible difference in the adhesion strength between the nematic and the isotropic phases of the LCE (broadly matching the tan δ data).

The study of the adhesion mechanism in LCEs^134^ also found an unusual dynamic phenomenon, making a strong connection with our understanding of polydomain nature of LCEs discussed earlier. There is always a certain “dwell time” (the time of contact of adhesive with the surface before the adhesion test is started) that is required to fully rearrange polymer chains and reach the maximum adhesion strength. However, in polydomain LCEs, this time has been remarkably long: the illustration in Figure 14(b) shows that the adhesion reaches the full strength only after 30 days of contact (this time strongly depends on the LCE parameters, e.g., higher cross-linking density will shorten this time but will also reduce the tackiness). However, if one sticks the LCE surface to a substrate and anneals it to the isotropic phase, then on natural cooling back to the nematic phase at ambient temperature the maximum adhesion strength is achieved straight away (see the “annealed” data set in Figure 14(b)).

This observation has formed the core of our current understanding of the LCE adhesion mechanism. We already discussed the nature of the polydomain state with random orientations of the nematic director, correlated only on the scale of 1–2 μm. Now consider what happens when such a material is strongly pressed against a rigid flat surface: the domains in the contact zone (and possibly several domain sizes into the material too) will be flattened against the surface and their director will be forced to lie in its plane, adjusting its orientation to the new pattern of stress. We know about the remarkably long time required for the rest of the domains to adjust their shape and orientation to comply with the forcefully flattened outer layer, and that is consistent with the long contact time observed.

On annealing, the elastomer becomes isotropic and then cools to form the new nematic domains that now grow with the flattened chains near the surface providing the additional internal boundary, that is, a new stress pattern different from the original stress-free equilibrium. The new domains form their director pattern in equilibrium with this boundary condition, which should be the same equilibrium state the nematic LCE was slowly relaxing toward if not annealed. Now, on peeling the surface off, the forcefully flattened boundary is no longer applied to the LCE, and the equilibrium domain structure should be the one formed naturally with no surface constraints due to the quenched random disorder. However, to reach that (the original stress-free equilibrium) texture, all the domains in the boundary layer must undergo a reverse deformation, with all the associated energy barriers to overcome. The faster the peeling, the stronger the barriers, as we have seen on the example of plateau stress at increasing deformation rates in Figure 11(a). Overcoming these barriers is the added work of adhesion, which we see in the pressure-sensitive adhesion of the LCE.

Investigations of anomalous LCE adhesion only started recently, and there is not yet an accepted consensus about the mechanism or the observations and certainly no theory. For instance, the arguments presented above heavily rely on the polydomain LCE nature and its manipulation, but what about an aligned monodomain LCE? In a recent study,^135^ this question was studied, and it was found that the peel strength (or the work of adhesion) is higher when the uniformly aligned director is along the contact line (i.e., perpendicular to the peel direction) and lower when the director is along the peel direction. Realizing that there is a high tensile stress perpendicular to the contact line (along the peel direction), one expects much lower stresses when the uniform director is perpendicular to that axis: this is a stripe-domain stretching geometry, as shown in Figure 9(a). Unfortunately, there was no comparison with the polydomain version of the same LCE, so it remains an open question whether the pressure-sensitive adhesion of polydomain LCE layer would be much stronger than that of a monodomain LCE in any orientation.

## Conclusions and Outlook

LCEs present three unique physical properties that make them an exciting “new state of matter”^6^ from the fundamental point of view, equally offering promise in “real world” applications.^91^ These are mechanical actuation, soft elasticity, and enhanced damping properties. The first (actuation) is the most studied and the most represented in the literature, being a mere reflection of the direct link between the mechanical shape of an elastomer and the underlying magnitude and orientation of its nematic order parameter. The other two are the properties of the nematic phase in the LCE, and they are fully reversible as the LCE is taken into the isotropic phase and back into the nematic phase again.

Although the stress-free changes of the natural shape on LCE actuation are spectacular, most of the applications of actuation are based on the local force delivered when the equilibrium shape change is blocked. Here, the most significant progress has been achieved during the 30 years of LCE research: taking the “blocking stress” as a comparative measure, Finkelmann’s first polysiloxane side-chain LCE actuators had this stress reaching only σ_*b*_ = 20 kPa^19^ in 1991, increasing to ca. 100 kPa^66^ in 2001. The first main-chain LCE of Percec had their blocking stress reaching ca. 2 MPa^14^ in 1992, which is about the same maximum to which the new thiol–acrylate main-chain LCE have reached in 2016.^136^ In recent years, there was a hard push to increase the mechanical strength of LCE while preserving at least some of their actuating capacity, and the most impressive levels of blocking stress have been achieved in the double-network LCE,^52,137^ of up to 40 MPa. In the meantime, the amazing range and versatility of 3D-printed LCE actuating structures has been dominating this field in the past few years.^101,138−140^

There are no meaningful applications of soft elasticity reported so far, but if we consider what could be possible, the answer must be in a setting when the shape of an elastomer is altered by external force but does not return back because there is no internal returning force along the soft deformation pathway. Utilizing this “fluid-like” behavior may not appear spectacular (e.g., any polymer gum would do the same), but with thermoset LCE the novelty is in their total shape recovery in the isotropic phase. Therefore, whatever shape alteration has been achieved and kept in the ambient state via the soft deformation pathway could be easily reset to the original natural shape by annealing.

In contrast, the applications of enhanced damping are obvious and versatile, both in the vibration-attenuation aspect and in pressure-sensitive adhesion. The exploration of these two physical effects is relatively new in the LCE field, and the intensity of their studies has not yet reached the high level of attention the LCE actuation has attracted, but in this author’s opinion they are more promising in the long run (and so their time will come very soon).

Importantly, the point needs to be reiterated that there is no adequate theoretical understanding of the enhanced vibration damping in LCEs, and this hampers their development (as well as the confusion about “dynamic soft elasticity” mentioned above). There are initial studies attempting to combine the nematic order dynamics with the rubber dissipation,^141,142^ but the problem is far from being solved. This understanding, to complement the equilibrium elasticity of the “Trace formula” with a proper model of anisotropic viscoelasticity, is needed to underpin and guide much of the current LCE research, which all focuses on the dynamics aspects of this system.

In materials, there is a need and a hope that LCE development would escape the current state of the art limitations imposed by the commercial availability of a few reacting monomers. It is important to maintain focus on “click” chemistry to continue the “democratic” nature of the field when almost anyone could make the LCE materials. However, staying within the same main-chain geometry is limited and restrictive. In particular, for many dynamics effects and applications, the side-chain LCE topology is likely to be preferable, yet so far only smectic side-chain LCEs were made using this thiol–acrylate chemistry with commercial reacting mesogens (while we have argued here that the nematic LCE phase is far more beneficial for dynamical effects). Finding suitable materials and demonstrating their application benefits would convince the commercial supplier providers to offer new supplies, so this remains another clear goal in future development. This author believes that we are standing on the verge of a new upward surge in the whole field, when all three of its elements (the theoretical understanding, the materials chemistry, and the application design) would experience an innovative push. This is an exciting time for those interested in liquid crystal elastomers and a promising ground for those planning to enter the field.

Finally, to conclude this brief overview, we may wish to take a page from the classical liquid crystal development story. After 40+ years of LC exploration, it was felt that in the early 2000s the field has saturated and excellent LCD screens populated consumer markets. Yet, researchers persisted and in recent years there have been a few breakthrough events, most notably the discovery of (long-predicted) nematic ferroelectrics. The potential in the discovery and applications of this new concept is only just emerging. In the LCE field, the original understanding has been that electric or magnetic fields are “too weak” to enact any meaningful shape changes of the cross-linked elastomer, yet considering a possibility of true ferroelectric nematic LCEs is breathtaking: here you might expect to generate electric power by a repetitive deformation (as in shoe soles in walking) or have a variety of mechanical sensors reporting directly to electric circuitry. Although the LCE field is mature now, it is very far from saturation and promises a long stretch of exciting discoveries.
